# Gene count estimation with pytximport enables reproducible analysis of bulk RNA sequencing data in Python

**DOI:** 10.1093/bioinformatics/btae700

**Published:** 2024-11-20

**Authors:** Malte Kuehl, Milagros N Wong, Nicola Wanner, Stefan Bonn, Victor G Puelles

**Affiliations:** Department of Clinical Medicine, Aarhus University, Palle Juul-Jensens Boulevard 99, Aarhus N, Midtjylland, 8200, Denmark; Department of Pathology, Aarhus University Hospital, Palle Juul-Jensens Boulevard 69, Aarhus N, Midtjylland, 8200, Denmark; Institute of Medical Systems Biology, University Medical Center Hamburg-Eppendorf, Falkenried 94, Hamburg, Hamburg, 20251, Germany; Center for Biomedical AI, University Medical Center Hamburg-Eppendorf, Martinistraße 52, Hamburg, Hamburg, 20246, Germany; Department of Clinical Medicine, Aarhus University, Palle Juul-Jensens Boulevard 99, Aarhus N, Midtjylland, 8200, Denmark; Department of Pathology, Aarhus University Hospital, Palle Juul-Jensens Boulevard 69, Aarhus N, Midtjylland, 8200, Denmark; III. Department of Medicine, University Medical Center Hamburg-Eppendorf, Martinistraße 52, Hamburg, Hamburg, 20246, Germany; Hamburg Center for Kidney Health, University Medical Center Hamburg-Eppendorf, Martinistraße 52, Hamburg, Hamburg, 20246, Germany; III. Department of Medicine, University Medical Center Hamburg-Eppendorf, Martinistraße 52, Hamburg, Hamburg, 20246, Germany; Hamburg Center for Kidney Health, University Medical Center Hamburg-Eppendorf, Martinistraße 52, Hamburg, Hamburg, 20246, Germany; Institute of Medical Systems Biology, University Medical Center Hamburg-Eppendorf, Falkenried 94, Hamburg, Hamburg, 20251, Germany; Center for Biomedical AI, University Medical Center Hamburg-Eppendorf, Martinistraße 52, Hamburg, Hamburg, 20246, Germany; Department of Clinical Medicine, Aarhus University, Palle Juul-Jensens Boulevard 99, Aarhus N, Midtjylland, 8200, Denmark; Department of Pathology, Aarhus University Hospital, Palle Juul-Jensens Boulevard 69, Aarhus N, Midtjylland, 8200, Denmark; III. Department of Medicine, University Medical Center Hamburg-Eppendorf, Martinistraße 52, Hamburg, Hamburg, 20246, Germany; Hamburg Center for Kidney Health, University Medical Center Hamburg-Eppendorf, Martinistraße 52, Hamburg, Hamburg, 20246, Germany

## Abstract

**Summary:**

Transcript quantification tools efficiently map bulk RNA sequencing (RNA-seq) reads to reference transcriptomes. However, their output consists of transcript count estimates that are subject to multiple biases and cannot be readily used with existing differential gene expression analysis tools in Python.

Here we present pytximport, a Python implementation of the tximport R package that supports a variety of input formats, different modes of bias correction, inferential replicates, gene-level summarization of transcript counts, transcript-level exports, transcript-to-gene mapping generation, and optional filtering of transcripts by biotype. pytximport is part of the scverse ecosystem of open-source Python software packages for omics analyses and includes both a Python as well as a command-line interface.

With pytximport, we propose a bulk RNA-seq analysis workflow based on Bioconda and scverse ecosystem packages, ensuring reproducible analyses through Snakemake rules. We apply this pipeline to a publicly available RNA-seq dataset, demonstrating how pytximport enables the creation of Python-centric workflows capable of providing insights into transcriptomic alterations.

**Availability and implementation:**

pytximport is licensed under the GNU General Public License version 3. The source code is available at https://github.com/complextissue/pytximport and via Zenodo with DOI: 10.5281/zenodo.13907917. A related Snakemake workflow is available through GitHub at https://github.com/complextissue/snakemake-bulk-rna-seq-workflow and Zenodo with DOI: 10.5281/zenodo.12713811. Documentation and a vignette for new users are available at: https://pytximport.readthedocs.io.

## 1 Introduction

Bulk RNA sequencing (RNA-seq) is a widely used tool to analyze differential gene expression across conditions (Stark *et al.* 2019). Thanks to the ever-decreasing cost of such experiments (Hu *et al.* 2021), it has become a mainstay in the research of many pathologies. Over the past decade, efficient transcript quantification tools that map reads from short-read RNA-seq to annotated transcriptomes have emerged (Zhang *et al.* 2017). While the aim of RNA-seq and transcript quantification software such as kallisto (Bray *et al.* 2016), Salmon (Patro *et al.* 2017), and RSEM (Li and Dewey 2011) is to detect and quantify RNA transcripts, such data are typically generated to infer gene-level expression from the corresponding transcripts and perform differential gene expression analysis (Love *et al.* 2016, Yi *et al.* 2018). However, transcript count estimates cannot be used out of the box for differential gene expression analysis as they require prior gene count quantification and correction for technical and biological biases. Importantly, differences in isoform usage between conditions may lead to inaccurate gene-level quantification without adjustments (Trapnell *et al.* 2013). Transcript counts are further subject to inferential uncertainty, since short reads may only map ambiguously to the respective reference transcriptome, resulting in skewed relative isoform abundances and an increased false-positivity rate when performing differential expression analysis (Pimentel *et al.* 2017, Sarantopoulou *et al.* 2021). To alleviate this problem, Salmon and kallisto optionally use bootstrapping or Gibbs sampling to sample the distribution of possible inferential replicates (Bray *et al.* 2016, Patro *et al.* 2017). However, many downstream analysis tools like PyDESeq2 (Muzellec *et al.* 2023) do not make use of this data. Thus, a flexible tool is needed that can quantify gene-level expression from transcript-level quantification files, optionally correct gene length and isoform usage bias, and utilize information from technical replicates.

Soneson *et al.* (2016) previously published tximport, an R package that addresses these issues. While many workflows for both differential gene expression analysis (Stark *et al.* 2019) and differential transcript usage analysis (Love *et al.* 2018) have been built on top of tximport, these have so far solely been available to the part of the bioinformatic community that employs the R programming language. Next to R, the Python programming language is widely used and counts with a growing ecosystem of RNA-seq analysis software, including Python versions of decoupleR (Badia-i-Mompel *et al.* 2022) and PyDESeq2 (Muzellec *et al.* 2023), a Python port of the widely-used DESeq2 package (Love *et al.* 2014). Together with advances in the single-cell RNA-seq field, these tools have come to form the scverse ecosystem of Python tools for omics analyses (Virshup *et al.* 2023). Given the well-established Python scientific computing ecosystem, it seems of interest to make the advances introduced by tximport available to the Python developer community, too.

Here we introduce pytximport, a Python implementation of the tximport package for bias-corrected gene count estimation from transcript-level abundances (Soneson *et al.* 2016). pytximport can process a multitude of input formats, is highly configurable, extensible, and its output is identical to tximport given the same parameters. pytximport is open source and can be installed from source or via the PyPi and Bioconda package registries. In addition to its core functionality, pytximport includes utility functions to generate transcript-to-gene mappings and to filter and process count matrices. Finally, we present an orchestrated end-to-end workflow for RNA-seq analysis, from FASTQ files to extensive downstream exploration. This workflow is based on Bioconda and scverse ecosystem tools and is provided as an easily adaptable, configurable, and customizable blueprint for Python users wishing to perform RNA-seq analysis.

## 2 Implementation

### 2.1 Code structure

**Figure 1.**
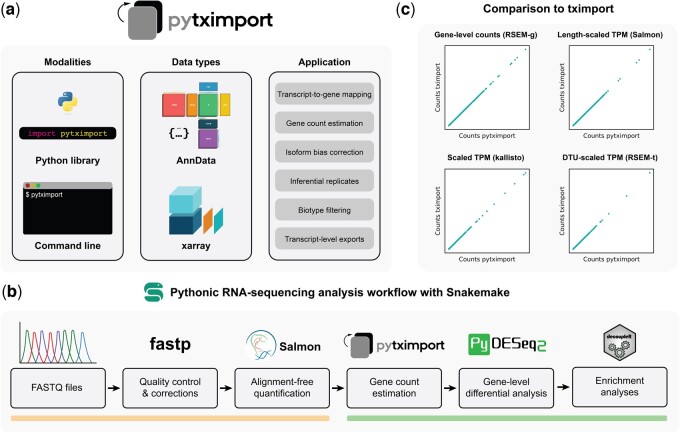
Overview of the pytximport package and its associated RNA-seq workflow. (a) pytximport package. pytximport is available for use as a Python library or from the command line. It can be configured to either output AnnData objects for integration with other scverse ecosystem software or xarray datasets. Common applications for pytximport include gene count estimation from transcript quantification files, isoform-usage bias correction, filtering of transcript-level data, and creation of transcript-to-gene mappings. (b) Pythonic RNA-seq analysis workflow. We propose a reproducible RNA-seq analysis workflow based on command-line software available through Bioconda (left line: Snakemake, fastp, Salmon) and scverse ecosystem Python packages (right line: pytximport, PyDESeq2, decoupleR). (c) Comparison with tximport. Counts from pytximport match counts from tximport exactly across different quantification modes and input files from different transcript quantification tools. RSEM-g: RSEM gene-level input; RSEM-t: RSEM transcript-level input. The Python logo is in the public domain and was provided through Bioicons. The AnnData logo is licensed under the BSD 3-Clause License. The xarray logo is provided under the Apache License Version 2.0. The Snakemake and PyDESeq2 logos are licensed under the MIT License. The Salmon and decoupleR logos are licensed under the GNU General Public License Version 3.

pytximport (Fig. 1a) is a modular, extensively annotated, unit-tested, and fully typed Python package. To facilitate the concurrent editing of metadata and the grouping of multiple named matrices (i.e. counts, abundances, and lengths), pytximport utilizes xarray (Hoyer and Hamman 2017) for efficient computation. The software is designed to be compatible with other packages in the scverse ecosystem of which it is a part (Virshup *et al.* 2023), relying on AnnData (Virshup *et al.* 2021) as a shared format.

### 2.2 Documentation

pytximport features comprehensive online documentation that guides new users through the entire workflow, from creating a transcript-to-gene mapping to configuring the import of quantification files and performing differential gene expression analysis.

The online documentation can be accessed at: https://pytximport.readthedocs.io

### 2.3 Similarities and differences between pytximport and tximport

pytximport faithfully implements the algorithm for gene-count estimation from transcript abundances introduced by tximport and described in detail by Soneson *et al.* (2016). It supports all RNA-seq input formats supported by the original implementation, including quantification files generated by Salmon/Sailfish (Patro *et al.* 2014, 2017), kallisto (Bray *et al.* 2016), piscem-infer (He *et al.* 2023), Oarfish (Jousheghani and Patro 2024), and RSEM (Li and Dewey 2011). In addition to this common functionality, pytximport can be configured to import quantification data from any tool, whose output can be represented as tab-separated values containing transcript ids, transcript counts, and effective transcript lengths. Conversely, tximport further supports alevin single-cell RNA-sequencing input files.

The core feature of both pytximport and tximport is the gene-level summarization of transcript counts, which requires a transcript-to-gene mapping. While tximport-based workflows typically rely on R packages such as GenomicFeatures (Lawrence *et al.* 2013) to create such mappings, pytximport includes utility functions that can generate mappings based on BioMart resources (Smedley *et al.* 2009) or user-provided annotation files.

To ensure accurate assignment of multimapping RNA reads, it can be advantageous to map reads to unrestricted references through decoy-aware selective alignment to genome-augmented transcriptomes (Srivastava *et al.* 2020). However, depending on the biological hypothesis, researchers may only be interested in a subset of genes, e.g., protein-coding genes. To allow for such post-hoc restrictions, pytximport optionally accepts a list of gene biotypes and subsets its results accordingly.

In order to interface with downstream analysis software within the scverse ecosystem, pytximport exports its results as AnnData objects (Virshup *et al.* 2021) by default. To increase its accessibility, pytximport can also be used through the command line. An overview of shared features and differences between pytximport and tximport is provided in Supplementary Table S1.

### 2.4 Python-centric RNA-seq analysis workflow

To facilitate the adoption of pytximport and provide Python developers with a blueprint for RNA-seq analysis, we offer a reproducible, end-to-end workflow (Fig. 1b) consisting of Bioconda software and scverse ecosystem Python packages orchestrated with Snakemake (Köster and Rahmann 2012). In a first step, we leverage fastp (Chen 2023) to assess read quality and trim FASTQ files. Then, sequence reference files are downloaded from Ensembl (Harrison *et al.* 2024) and processed by Salmon (Patro *et al.* 2017) along with the trimmed read data to quantify transcript counts via selective alignment. Next, we employ pytximport to generate isoform bias-corrected gene-level count estimates based on these transcript counts. Lastly, we use PyDESeq2 (Muzellec *et al.* 2023) together with the Python version of decoupleR (Badia-i-Mompel *et al.* 2022) to identify differentially expressed genes and perform gene set enrichment analysis as well as transcription factor activity analysis using the Reactome pathway database (Milacic *et al.* 2024) and the CollecTRI transcription factor network resource (Müller-Dott *et al.* 2023).

The workflow is available via GitHub at: https://github.com/complextissue/snakemake-bulk-rna-seq-workflow

### 2.5 Evaluation on RNA-seq data

We evaluate pytximport as part of the Python-centric RNA-seq analysis workflow on a publicly available dataset with two groups of 12 replicates each. Details regarding the data source and processing steps are provided in the Supplementary Online Methods.

## 3 Results

### 3.1 Comparison with tximport

We compared the outputs of pytximport with tximport over several configurations, including gene-level counts, gene-level length-adjusted library size-scaled transcripts per million (TPM), transcript-level library size-scaled TPM, and transcript-level median gene-length-adjusted library size-scaled TPM. This comparison revealed exact agreement for all counts, abundances and lengths for all configurations and input data sources (Fig. 1c), confirming correct implementation and extending the benefits offered by tximport to researchers who may choose to use Python.

### 3.2 Performance benchmark

To quantify computational efficiency, we compared the different quantification modes of pytximport and tximport across quantification files from Salmon (Patro *et al.* 2017), kallisto (Bray *et al.* 2016), and RSEM (Li and Dewey 2011), measuring single-core wall time requirement. Depending on the configuration, the median runtime of pytximport ranged from 1.0 to 24.4 s. Similarly, tximport had a median time requirement between 2.2 and 27.1 s (Supplementary Table S2). Although pytximport improved performance for the configurations tested, both implementations had a negligible footprint in the context of the end-to-end RNA-seq analysis workflow and seem well suited for use within their respective ecosystems.

## 4 Conclusion

In summary, pytximport provides a reliable Python package for isoform bias-corrected gene count estimation from transcript abundances from a variety of transcript quantification tools, thereby efficiently preparing the data for further downstream processing and seamlessly integrating with Python packages for differential gene expression analysis, such as PyDESeq2.

The release of pytximport enables Python developers and command-line users alike to run pipelines that previously required knowledge of the R programming language. Because pytximport is open source, its modular architecture, extensive documentation, and configurability will allow developers to build on it and further enrich the Python omics ecosystem.

## Supplementary Material

btae700_Supplementary_Data

## Data Availability

The public IgA nephropathy dataset used in the evaluation of pytximport is available via the European Nucleotide Archive with accession: PRJNA593015.
