# Supplementary material for: Gene count estimation with pytximport enables reproducible analysis of bulk RNA sequencing data in Python
Source: Bioinformatics. 2024 Nov 20;40(12):btae700. doi: 10.1093/bioinformatics/btae700 (PMC11629965; doi:10.1093/bioinformatics/btae700)
Supplement: btae700_Supplementary_Data [file btae700_supplementary_data.pdf]

Supplementary material for online publication

Gene count estimation with pytximport enables reproducible analysis of bulk RNA sequencing data in Python

Supplementary online methods

Pythonic RNA sequencing analysis workflow

Snakemake (v8.16.0; Köster and Rahmann 2012) was installed from the Bioconda repository (Grüning *et al.* 2018) through the Mamba package manager. Further computational processing was divided into the following steps, all of which were orchestrated with Snakemake: quality control and trimming of FASTQ files, download of reference files, read quantification, gene-level count estimation, differential gene analysis, and gene set enrichment analysis.

Quality control and trimming of FASTQ files

This step used the Snakemake wrappers (v4.3.0) for FastQC (Andrews 2023), MultiQC (Ewels *et al.* 2016) and fastp (Chen 2023). First, all read files were individually assessed with FastQC and reports were aggregated with MultiQC. Next, paired-end read files were processed with fastp and low quality score reads as well as remaining adapter sequences were removed.

Download of reference files

The Ensembl (Harrison *et al.* 2024) human/murine reference genome, transcriptome and annotation (release 112) were downloaded using the Snakemake Ensembl wrapper (v4.3.0). Next, pytximport (v0.10.0) was used to generate a transcript-to-gene mapping based on the Ensembl release 112 available through BioMart (Smedley *et al.* 2009).

Read quantification via selective alignment

This step made use of the Salmon (Patro *et al.* 2017) Snakemake *decoy*, *index*, and *quant* wrappers (v4.3.0). First, the transcriptome and the genome were concatenated to form a joined reference for decoy-aware mapping. Then, a Salmon index was built based on this gentrome. Lastly, the Salmon *quant* wrapper was employed to quantify transcript counts from the fastp-processed read files, using selective alignment with 48 bootstraps and GC-bias correction.

Gene-level count estimation

The output from Salmon was aggregated with pytximport (v0.10.0) using the transcript-to-gene mapping built from the reference annotation. Counts and abundances were first recalculated at the sample levels by setting all transcript counts to their respective median count across all Salmon bootstraps. Then, counts, abundances and lengths were summarized at the gene-level, missing gene lengths were inferred, and counts were recomputed from abundances with gene length-aware library-size scaling of TPM (*length_scaled_tpm*). Results were exported to an AnnData file.

Differential gene expression analysis

Gene-level count estimates were filtered to include only genes with a minimum mean count of 20 across all samples, a minimum count of 50 in at least one sample, and a minimum count of 20 in at least two samples. Differential gene expression analysis was performed using PyDESeq2 (v0.4.10; Muzellec *et al.* 2023) with default settings and $\log_2$ fold-change shrinking. Genes were considered differentially expressed if their $\log_2$ fold change was greater than 0.5 or lower than -0.5 and their false discovery rate-adjusted p-value was less than 0.01.

Gene set enrichment analysis

All gene set enrichment analyses were performed with decoupler-py (v1.7.0; Badia-i-Mompel *et al.* 2022). First, the CollecTRI transcription factor network resource (Müller-Dott *et al.* 2023) was downloaded, and transcription factor activity profiles were determined using decoupler's univariate linear modelling function, *run_ulm*. Next, the likely importance of conserved pathways was evaluated using the PROGENy (Schubert *et al.* 2018) reference

and decoupler's multivariate linear modeling function, *run_mlm*. Lastly, REACTOME pathway networks (Milacic *et al.* 2024) were loaded from the MSigDb (v2023.2) reference provided by Omnipath (Türei *et al.* 2021) and overrepresentation analysis was performed based on the statistically significantly differentially expressed genes with decoupler's *get_ora_df* function.

Comparison to tximport

We compared pytximport and tximport across a great variety of configurations on a publicly available RNA-seq dataset with 24 samples, representing 12 replicates per condition.

Data source

Immunoglobulin A nephropathy (IgAN) is a common form of glomerulonephritis in which mesangial immunoglobulin A deposits trigger an inflammatory and proliferative response, ultimately leading to loss of kidney function (Stamellou *et al.* 2023). To benchmark pytximport and the Pythonic Snakemake workflow, we tested it on a public dataset of microdissected glomeruli from patients with IgAN or controls (Park *et al.* 2020).

Data generation

Transcript quantification files were generated as part of the Snakemake workflow described above. To compare the implementations across a wider range of tools, we extended the read quantification step to also include RSEM (v1.3.3; Li and Dewey 2011) with Bowtie2 (v2.5.4;Langmead and Salzberg 2012) as well as the Snakemake wrapper (v4.3.0) for kallisto (Bray *et al.* 2016) which we ran with 48 bootstraps. This generation step was applied to all samples from the public RNA-seq dataset described above. The Snakemake workflow updated to use multiple read mapping tools is available through GitHub: https://github.com/complextissue/snakemake-bulk-rna-seq-workflow/tree/multiple-aligners

Count matrix generation

For all transcript-level input files from the different quantification tools, we generated 3 different gene-level count matrices by setting the *counts_from_abundance* parameter of pytximport (v0.10.0) and the *countsFromAbundance* parameter of tximport (v1.30.0) to None / "no", "length_scaled_tpm" / "lengthScaledTPM" and "scaled_tpm" / "scaledTPM" respectively. Similarily, we generated 3 transcript-level count matrices by setting the *counts_from_abundance* parameter of pytximport and the *countsFromAbundance* parameter of tximport to None / "no", "scaled_tpm" / "scaledTPM" and "dtu_scaled_tpm" / "dtuScaledTPM". For Salmon and kallisto input files, 2 different sets of counts matrices were generated, one that did not make use of inferential replicates and another one based on the median transcript counts across all inferential replicates of the respective transcript. Gene-level input files from RSEM were also processed to generate gene count matrices.

Confirmation of agreement

For each comparison, the counts generated by pytximport and tximport were read from the corresponding .csv files in which they were saved. Full agreement between pytximport and tximport was ensured by using the *assert_frame_equal* function from the testing submodule of the pandas software package (v2.2.2; McKinney 2011) with *atol* set to *1e-6* to account for the imprecision of floating point numbers. To visualize the agreement, we plotted the counts generated by pytximport against the counts generated by tximport for select comparisons.

Performance benchmarking

All quantification files from the Immunoglobulin A nephropathy RNA-sequencing dataset generated as described above were processed with pytximport (v0.9.0) and tximport (v1.32.0). Benchmarking was performed on an AMD Ryzen Threadripper PRO 5965WX - 24-Core 3.80/4.50GHz central processing unit with 256 GB of memory, Python 3.12.6 and R 4.4.1. Programs were run inside the "condaforge/mambaforge" Docker container and limited to a single thread through the --cpuset-cpus configuration option. Wall time requirement was measured with pyperf for the Python scripts (v2.7.0) and bench for the R scripts (v1.1.3). To ensure a focussed comparison, recommended optional dependencies were installed and the transcript-to-gene mappings were preloaded. To

account for warm-up inaccuracies, each quantification was performed 7 times, and the median value was reported. The benchmarking scripts have been deposited on Zenodo: https://zenodo.org/records/13883208

Supplementary tables

Supplementary table 1: Similarities and differences between pytximport and tximport

**Feature**	**pytximport (v0.10.0)**	**tximport (v1.32.0)**
**Available interfaces**	Python, command line	R
**Software ecosystems**	scverse, Bioconda	Bioconductor
**(Online) documentation with examples**	Yes	Yes
**Supported quantification files**	Salmon, kallisto, RSEM, Sailfish, Oarfish, Stringtie, piscem-infer, .tsv files	Salmon, kallisto, RSEM, Sailfish, Oarfish, Stringtie, piscem-infer
**Gene-level count quantification modes**	Counts with offset matrix, library size-scaled gene-summarized transcripts per million, library size and gene-length scaled gene-summarized transcripts per million	Counts with offset matrix, library size-scaled gene-summarized transcripts per million, library size and gene-length scaled gene-summarized transcripts per million
**Transcript-level count quantification modes**	Counts with offset matrix, library size-scaled transcripts per million, library size-scaled median gene-length adjusted transcripts per million	Counts with offset matrix, library size-scaled transcripts per million, library size-scaled median gene-length adjusted transcripts per million
**Inferential replicates**	Yes	Yes
**Alevin single-cell RNA-sequencing input support**	No	Yes
**Transcript-to-gene mapping generation**	Yes, based on BioMart servers or gene transfer format annotation files	No, available through other R packages
**Biotype filtering**	Yes, if the biotype was included in the transcript id string	No
**Transcript version removal**	Yes, from both the imported data and the transcript-to-gene mapping	Yes, but only from the imported data
**Available end-to-end workflows**	Snakemake	Nextflow, Snakemake
**Output format**	AnnData object, xarray Dataset object, .h5ad file, .csv file, SummarizedExperiment (experimental)	R object, SummarizedExperiment available through *tximeta* (Love *et al.* 2020)

Supplementary table 2: Performance benchmarking

		pytximport	tximport
**Input format**	**Quantification mode**	**Time (s)**	**Time (s)**
**Salmon**	Transcript counts	**2.2**	4.3
	Transcript counts, library size scaled TPM	**2.3**	4.5
	Transcript counts, median gene-length and library size scaled TPM	**6.2**	8.1
	Gene counts	**3.1**	7.5
	Gene counts, library size scaled TPM	**3.2**	7.3
	Gene counts, gene length and library size scaled TPM	**3.3**	7.5
**Salmon with 48 inferential replicates**	Transcript counts	**14.5**	19.6
	Transcript counts, library size scaled TPM	**14.5**	19.7
	Transcript counts, median gene-length and library size scaled TPM	**18.6**	22.8
	Gene counts	**20.5**	24.0
	Gene counts, library size scaled TPM	**21.1**	24.0
	Gene counts, gene length and library size scaled TPM	**20.1**	24.7
**kallisto**	Transcript counts	**1.6**	2.0
	Transcript counts, library size scaled TPM	**1.6**	2.3
	Transcript counts, median gene-length and library size scaled TPM	**5.9**	6.0
	Gene counts	**2.7**	5.0
	Gene counts, library size scaled TPM	**2.6**	5.3
	Gene counts, gene length and library size scaled TPM	**2.6**	5.2
**kallisto with 48 inferential replicates**	Transcript counts	**16.8**	19.2
	Transcript counts, library size scaled TPM	**17.3**	19.4
	Transcript counts, median gene-length and library size scaled TPM	**21.5**	23.7
	Gene counts	**24.1**	26.5
	Gene counts, library size scaled TPM	**24.2**	27.1
	Gene counts, gene length and library size scaled TPM	**24.4**	26.5
**RSEM with transcripts-level input**	Transcript counts	**3.2**	8.0
	Transcript counts, library size scaled TPM	**3.2**	8.0
	Transcript counts, median gene-length and library size scaled TPM	**10.0**	12.5
	Gene counts	**4.3**	11.2
	Gene counts, library size scaled TPM	**4.6**	11.5
	Gene counts, gene length and library size scaled TPM	**4.7**	11.3
**RSEM with gene-level input**	Gene counts	**1.0**	2.2

All reported values represent median values across 7 runs when importing quantification files based on the public Immunoglobulin A nephropathy RNA-sequencing dataset.

References

Andrews S. s-andrews/FastQC. 2023.

Badia-i-Mompel P, Vélez Santiago J, Braunger J *et al.* decoupleR: ensemble of computational methods to infer biological activities from omics data. *Bioinforma Adv* 2022;**2**:vbac016.

Bray NL, Pimentel H, Melsted P *et al.* Near-optimal probabilistic RNA-seq quantification. *Nat Biotechnol* 2016;**34**:525–7.

Chen S. Ultrafast one-pass FASTQ data preprocessing, quality control, and deduplication using fastp. *iMeta* 2023;**2**:e107.

Ewels P, Magnusson M, Lundin S *et al.* MultiQC: summarize analysis results for multiple tools and samples in a single report. *Bioinformatics* 2016;**32**:3047–8.

Grüning B, Dale R, Sjödin A *et al.* Bioconda: sustainable and comprehensive software distribution for the life sciences. *Nat Methods* 2018;**15**:475–6.

Harrison PW, Amode MR, Austine-Orimoloye O *et al.* Ensembl 2024. *Nucleic Acids Res* 2024;**52**:D891–9.

Köster J, Rahmann S. Snakemake—a scalable bioinformatics workflow engine. *Bioinformatics* 2012;**28**:2520–2.

Langmead B, Salzberg SL. Fast gapped-read alignment with Bowtie 2. *Nat Methods* 2012;**9**:357–9.

Li B, Dewey CN. RSEM: accurate transcript quantification from RNA-Seq data with or without a reference genome. *BMC Bioinformatics* 2011;**12**:1–16.

Love MI, Soneson C, Hickey PF *et al.* Tximeta: Reference sequence checksums for provenance identification in RNA-seq. *PLOS Comput Biol* 2020;**16**:e1007664.

McKinney W. pandas: a foundational Python library for data analysis and statistics. *Python High Perform Sci Comput* 2011;**14**:1–9.

Milacic M, Beavers D, Conley P *et al.* The Reactome Pathway Knowledgebase 2024. *Nucleic Acids Res* 2024;**52**:D672–8.

Müller-Dott S, Tsirvouli E, Vazquez M *et al.* Expanding the coverage of regulons from high-confidence prior knowledge for accurate estimation of transcription factor activities. *Nucleic Acids Res* 2023;**51**:10934–49.

Muzellec B, Teleńczuk M, Cabeli V *et al.* PyDESeq2: a python package for bulk RNA-seq differential expression analysis. *Bioinformatics* 2023;**39**:btad547.

Park S, Yang SH, Jeong CW *et al.* RNA-Seq profiling of microdissected glomeruli identifies potential biomarkers for human IgA nephropathy. *Am J Physiol-Ren Physiol* 2020;**319**:F809–21.

Patro R, Duggal G, Love MI *et al.* Salmon provides fast and bias-aware quantification of transcript expression. *Nat Methods* 2017;**14**:417–9.

Schubert M, Klinger B, Klünemann M *et al.* Perturbation-response genes reveal signaling footprints in cancer gene expression. *Nat Commun* 2018;**9**:20.

Stamellou E, Seikrit C, Tang SCW *et al.* IgA nephropathy. *Nat Rev Dis Primer* 2023;**9**:1–21.

Türei D, Valdeolivas A, Gul L *et al.* Integrated intra- and intercellular signaling knowledge for multicellular omics analysis. *Mol Syst Biol* 2021;**17**:e9923.
